# Cauterization of Narrow Root Canals Untouched by Instruments by High-Frequency Current

**DOI:** 10.3390/ma16072542

**Published:** 2023-03-23

**Authors:** Hiromichi Kumagai, Tsutomu Sugaya, Toshihiko Tominaga

**Affiliations:** 1 Department of Periodontology and Endodontology, Faculty of Dental Medicine, Hokkaido University, N13W7, Kita-ku, Sapporo 060-8586, Hokkaido, Japan; 2 Tominaga Dental Clinic, Setotyomyoujinshitahonjo 197-3, Naruto 771-0360, Tokushima, Japan

**Keywords:** electrosurgery, electric impedance, cautery, root canal therapy

## Abstract

The mechanical removal of bacteria is fundamental to the treatment of infected root canals, but complete sterilization of biofilms tends not to extend to uninstrumented areas. However, during electrical conduction to a root canal filled with a conductor, the higher impedance where the root canal is narrower generates Joule heat that may result in a large temperature increase and sterilization. The effect of a high-frequency electric current on the wall of a simulated narrow root canal was investigated by scanning electron microscopy (SEM) and energy dispersive X-ray spectrometry (EDS). Simulated root canals, 0.1 mm in diameter, were prepared in dentine blocks. The root canal wall was treated with Plank-Rychlo solution for 5 min to create a decalcified layer. The simulated root canal was filled with either saline or NaClO, and 150 or 225 V at 520 kHz was applied for 0 s, 1 s, or 5 s. As the conduction time increased, and when the saline was replaced with NaClO, the proportion with a flat decalcified surface decreased, dentinal tubules and a lava-like morphology were significantly more evident on SEM (*p* < 0.01), and EDS showed significant decreases in carbon and oxygen and increases in calcium (*p* < 0.01). It was concluded that filling uninstrumented root canals with NaClO and using electrical conduction for 5 s could incinerate and eliminate the organic material of the root canal wall. The application of high-frequency electric current may lead to the cure of many cases of persistent apical periodontitis.

## 1. Introduction

The removal of bacteria, their products, and other pathogenic factors is fundamental to the treatment of infected root canals, which are cured by appropriate root canal treatment in approximately 90% of cases [1,2]. Nickel–titanium files have improved the technical quality of root canal shaping and the cleaning effect, enhancing the clinical outcome of root canal treatment [3,4,5,6,7,8,9]. However, in root canal treatment of teeth with a calcified canal, ledge, step, or other obstruction of the root canal, the instruments fail to touch the root canal wall, and the treatment outcomes are not as good [10,11,12,13,14]. Root canal irrigation with NaClO and intracanal medication with calcium hydroxide has long been used to eradicate pathogenic factors in the vicinity of the apex in the root canal [15,16,17]. Novel root canal irrigants, irrigation devices, and intracanal medication have also been studied [18,19,20,21,22,23]. However, their effectiveness tends not to extend to uninstrumented areas, and complete sterilization of biofilms is considered difficult [24,25,26,27]. In addition, new technological innovations are effective for the prevention and treatment of fragile and uncooperative patients, such as autism spectrum disorder patients [28]. Root canal treatment in particular takes a long time, so the development of a rapid, minimally invasive, and highly effective method would be of great help in the treatment of such patients.

Apicoectomy is commonly performed when sterilization fails and inflammation occurs in the apical periodontal tissue. Endodontic periapical surgery with a microscope has a high success rate of greater than 90% [29,30], but it is highly invasive and sometimes cannot be performed due to anatomical or systemic reasons. Tooth extraction is indicated if inflammation recurs after surgery. Lateral canals and apical ramifications have been observed in 75% of teeth after surgical removal of apical roots [25], most of which have been filled with bacteria. Therefore, sterilization of uninstrumented areas appears to significantly contribute to improving the success rate of endodontic therapy.

High-frequency electric current has long been used for the electrosurgery of soft tissue [31,32]. Studies of the use of high-frequency currents in root canal treatment by using the heat generated from the electrical contact resistance between the electrode tip and the pulp or root canal wall [33,34] have investigated pulp coagulation [35,36,37] and sterilization of the inside of the root canal [38]. However, during electrical conduction to the root canal, the higher impedance at the apical constriction and in accessory canals where the root canal is narrower means that the thermal energy increases in accordance with Ohm’s law and Joule’s law [39], generating Joule heat that may result in a large temperature increase. In a numerical simulation, Tarao et al. [40] found that passing a 500 kHz, 60 V electric current through a root canal with an apical foramen 0.4 mm in diameter resulted in a 62 °C increase in the vicinity of the apical foramen after 0.1 s. Accordingly, when apical patency fails because of a ledge, step, or other obstruction, and the uninstrumented root canal is narrow, the current density of a high-frequency electric current increases, and Joule heat is generated, which may make it possible to cauterize the pathogenic factors in the root canal. However, all previous studies of the application of a high-frequency electric current in root canal treatment have used it to heat the tip of the file, and none have investigated its effect at the apical constriction where the temperature increase is the greatest.

The null hypothesis was that the conduction of high-frequency current to an uninstrumented root canal does not cauterize the organic matter of the root canal wall and does not melt the dentine. Whether root canal fluid and the voltage and conduction time of high-frequency current to simulated root canals affect the disappearance of the organic material on the root canal wall and the melting of dentine were evaluated in this study.

## 2. Materials and Methods

### 2.1. Root Canal Model Preparation

**Figure 1:**
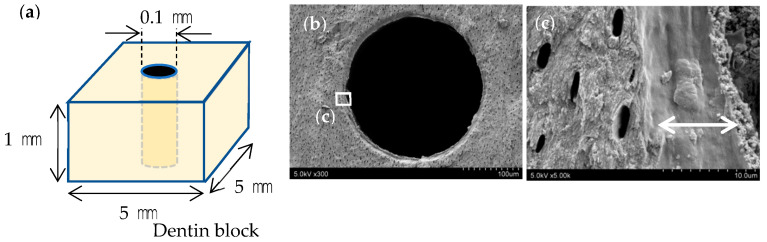
Preparation of simulated root canals. (**a**) Perforation of the dentine block with a diameter of 0.1 mm (**b**) SEM image of the fractured simulated root canal after decalcification. (**c**) Decalcified layer measuring approximately 10 μm in its dry condition (arrows).

A turbine (Twin power turbine ultra M, J. Morita Manufacturing, Kyoto, Japan) under water irrigation at 400,000 rpm and a diamond point (DIA-Burs CE-12F, Mani, Utsunomiya, Japan) were used to prepare 54, 1 mm thick, dentine blocks, with length and width of 5 mm each, from frozen bovine teeth, and the surfaces were polished with #2000 waterproof abrasive paper. A #10 engine driven reamer (Mani) and a motor (TriAuto ZX2, 800 rpm, torque 4 Ncm, J. Morita Manufacturing) were then used to perforate each block without irrigation to produce a simulated root canal approximately 0.1 mm in diameter (Figure 1a), which was decalcified with Plank-Rychlo solution (7.0% aluminum chloride, 8.5% hydrochloric acid, and 5.0% formic acid) for 5 min to prepare a decalcified layer in the simulated root canal wall (Figure 1b,c). A polypropylene tube and 4-META/MMA-TBB resin (Super-Bond, Sun Medical, Moriyama, Japan) were used to create a surrounding wall approximately 3 mm in height around each dentine block.

### 2.2. Conduction of High-Frequency Electric Current

**Figure 2:**
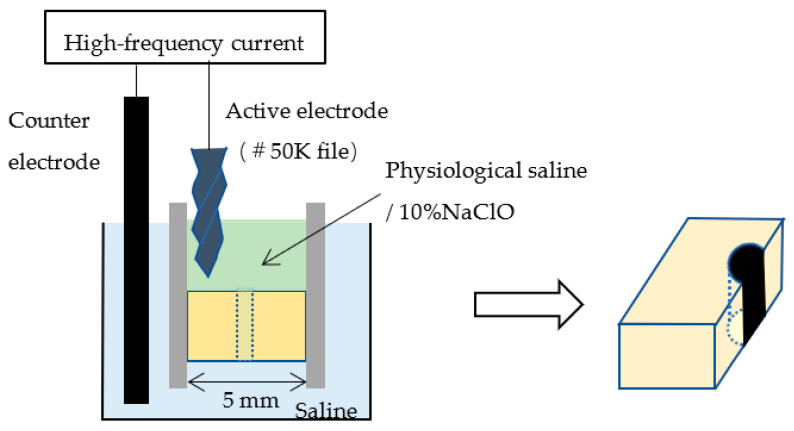
Electrical conduction to the simulated root canal and cutting through the block.

The bottom surface of the dentine block was placed in contact with physiological saline (0.9% NaCl), and the surrounding wall on the top and the simulated root canal were filled with either physiological saline or 10% NaClO. A stainless steel #50K file (Mani) was used as the active electrode, and this was placed in contact with the upper surface of the dentine block at a distance of at least 1 mm from the simulated root canal. The counter electrode was placed in the physiological saline (Figure 2). A high-frequency generator (J. Morita Manufacturing) was used to generate a high-frequency (520 kHz) current; duty 70%; voltage of 150 V or 225 V; and electrical conduction time of 0 s, 1 s, or 5 s (Table 1). Controls through which a current was not passed were filled with 10% NaClO and rinsed with water after 10 s. After electrical conduction, the dentine block was cut through with a turbine under water irrigation at 400,000 rpm (Twin power turbine ultra M, J. Morita Manufacturing) and a diamond point (DIA-Burs CE-12F, Mani) to expose the simulated root canal (Figure 2).

### 2.3. Evaluation Method

#### 2.3.1. Evaluation by Optical Microscopy

**Figure 3:**
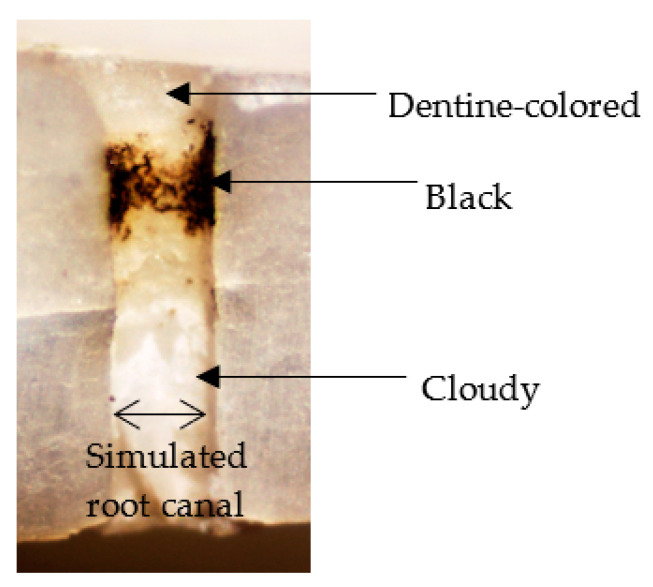
Classification of simulated root canal wall by discoloration.

Three randomly chosen sites, one each from the upper, middle, and lower simulated root canal wall, were examined with an optical microscope. The root canal wall was classified as dentine-colored, cloudy, or black, depending on the discoloration seen (Figure 3).

#### 2.3.2. Evaluation by Scanning Electron Microscopy (SEM)

**Figure 4:**
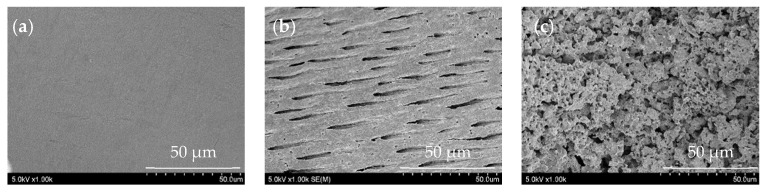
Classification of simulated root canal walls by morphology. (**a**) Flat surface, (**b**) dentinal tubular surface, and (**c**) lava-like surface.

A platinum–palladium sputtered coating was applied with an ion-sputtering device (E1030, Hitachi, Tokyo, Japan), and secondary electron images of three randomly chosen sites, one each from the upper, middle, and lower simulated root canal walls, were examined with a scanning electron microscope (S4800, Hitachi) at an accelerating voltage of 5.0 kV and 1000× magnification. The morphology that occupied the majority of the microscopic field was classified as a flat surface; a dentinal tubular surface with exposed dentinal tubules; or a superficially rough, porous lava-like surface (Figure 4).

#### 2.3.3. Evaluation by Energy Dispersive X-ray Spectroscopy

**Figure 5:**
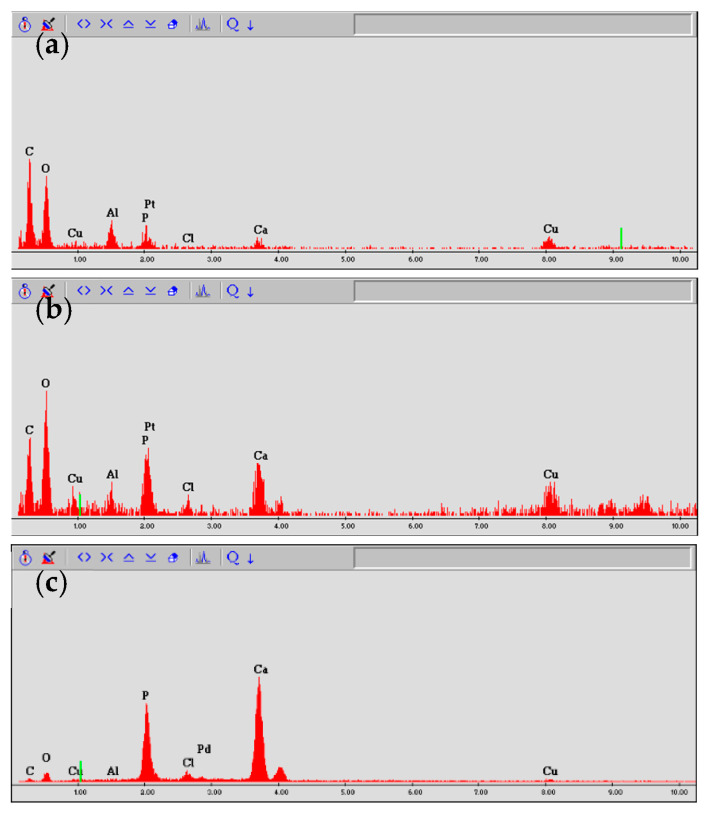
Classification of simulated root canal walls by EDS spectrum. (**a**) Decalcified surface, (**b**) dentine surface, and (**c**) mineral surface.

Elemental analysis of three randomly chosen sites, one each from the upper, middle, and lower simulated root canal walls, was conducted via energy dispersive X-ray spectrometry (EDS) using a scanning electron microscope (S2380N, Hitachi) equipped with an EDS spectroscope (Si (Li) semiconductor detector, Genesis CDU, EDAX Japan, Tokyo) with an acceleration voltage of 10 kV in spot scan mode. On the basis of their spectral patterns, they were classified into one of the following three categories: (i) decalcified surface containing large amounts of carbon and oxygen, but little calcium; (ii) dentine surface containing carbon, oxygen, and calcium; or (iii) mineral surface containing calcium, but little carbon or oxygen (Figure 5).

#### 2.3.4. Statistical Analysis

The data were analyzed by the χ^2^ test using IBM SPSS Statistics 21 (IBM Japan, Tokyo, Japan).

## 3. Results

### 3.1. Evaluation by Optical Microscopy

**Figure 6:**
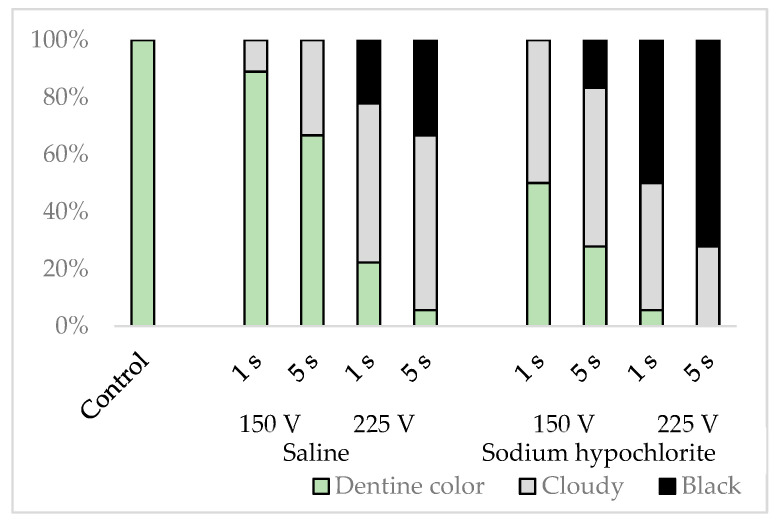
Classification results of simulated root canal walls by discoloration.

The simulated root canal walls in the control group were dentine-colored with no discoloration, but those in the electrified group had walls in varying states of discoloration, with no specific discoloration evident in any particular part of the root canal. The discoloration varied depending on the solution in the root canal, the electrification time, and voltage (*p* < 0.001), with more cloudiness and blackness generated by sodium hypochlorite than by physiological saline, by a voltage of 225 V than by a voltage of 150 V, and by electrification for 5 s than by electrification for 1 s (Figure 6).

### 3.2. Evaluation by SEM

**Figure 7:**
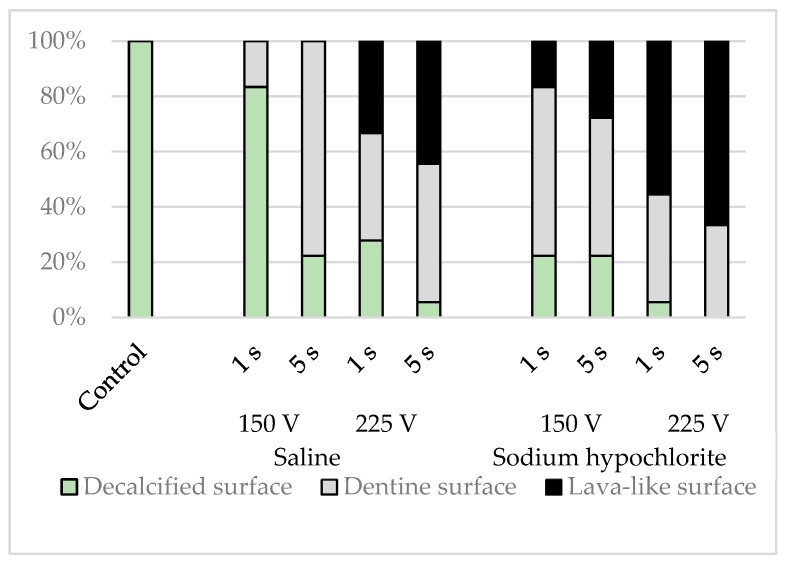
Classification results of simulated root canal walls by morphology.

The simulated root canal walls in the control group were all flat, whereas the electrified group exhibited a range of simulated root canal wall morphologies. Some samples contained a mixture of flat surfaces, surfaces containing dentinal tubules, and lava-like surfaces, but these morphological changes varied depending on the solution in the root canal, the electrical conduction time, and the voltage (*p* < 0.001), with more dentinal tubular and lava-like surfaces generated by sodium hypochlorite than by physiological saline, by a voltage of 225 V than by a voltage of 150 V, and by electrical conduction for 5 s than by electrical conduction for 1 s (Figure 7).

### 3.3. Evaluation by EDS

**Figure 8:**
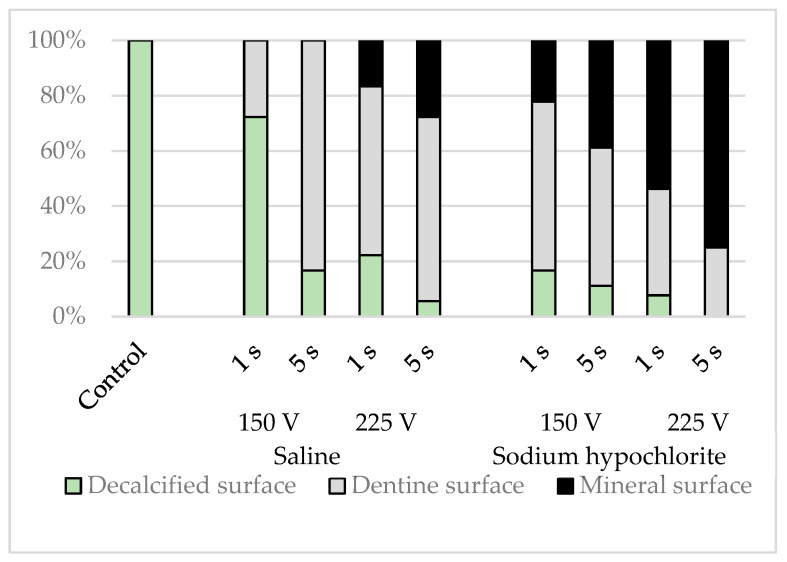
Classification results for simulated root canal walls by elemental analysis.

EDS elemental analysis of the simulated root canals showed that the intensities of calcium were lower in the control group, whereas those of carbon and oxygen were higher. The spectral patterns of the simulated root canals after electrical conduction varied depending on the solution in the root canal, the conduction time, and the voltage (*p* < 0.001), with carbon and oxygen decreased and calcium increased to a greater extent by sodium hypochlorite than by physiological saline, by a voltage of 225 V than by a voltage of 150 V, and by conduction for 5 s than by conduction for 1 s; most spectral patterns indicated lower levels of carbon and oxygen and higher levels of calcium (Figure 8).

The sites that appeared flat on SEM had far lower intensities of calcium. Carbon, oxygen, and calcium were all detected in the dentinal tubular surfaces. In the lava-like surfaces, the intensities of carbon and oxygen decreased, and those of calcium increased. There was no site at which carbon alone was strongly detected.

## 4. Discussion

In this study, the simulated root canal walls were decalcified with Plank-Rychlo solution for 5 min, and in their dry state, a homogeneous layer, approximately 10 μm thick, was formed. Because Planck-Rychlo solution quickly demineralizes and causes less swelling and shrinkage, it was thought to be suitable for creating an organic layer on the root canal wall. The decrease in the intensities of calcium seen on elemental analysis also suggested that decalcification caused the formation of a layer of organic material in the root canal wall. Because the thick organic material layer formed by decalcification dried and contracted during the preparation of the SEM samples, it became a flat surface covering the dentinal tubules. Because this did not become detached from the root canal wall during specimen preparation, this may be an effective method for investigating whether electrical conduction eliminates organic material from the root canal wall. Physiological saline and 10% NaClO were used as the root canal fluid during energization. The reason why the highest concentration of NaClO for root canal irrigation available was selected in this study was that a high-concentration solution would increase conductivity, and increased current density might enhance the elimination of organic material. Because there are no data showing the superiority of highly concentrated NaClO, the relationship between concentration and the conduction effect is a future aspect to examine.

To demonstrate that the organic material on the root canal wall was eliminated and the dentine was exposed, it was necessary to examine whether carbon and oxygen decrease and calcium increases. Carbon coating for EDS might modify the carbon and result in unclear results. Therefore, Pt-Pd coating was performed in this study. At the sites where the dentinal tubules were exposed on SEM observation after electrical conduction, EDS analysis showed the presence of carbon, calcium, and oxygen, suggesting that the layer of organic material on the simulated root canal walls had been eliminated and that what was detected was dentine organic matrix and hydroxyapatite. Al and Cu were faintly detected; the Al may be the residue of the components contained in the Planck-Rychlo solution, and the Cu may be the residue of the engine-driven reamer used when preparing the artificial root canal. Although the EDS evaluation was qualitative in the present study, the morphological evaluation with SEM and the elemental analysis with EDS were generally consistent. From these comprehensive evaluations, the elimination of organic material on the root canal wall was assessed with reasonable accuracy.

The surface of the root canal wall after removal of organic material resembles the dentine surface after Er-YAG laser treatment [41,42]. With Er-YAG lasers, the light energy absorbed by water and organic components becomes heat energy, causing photothermal evaporation, vaporization and microexplosions, and the removal of water-containing tissue [43,44]. Electrocautery has a high current density around the electrodes, and the water in the soft tissue rapidly evaporates due to Joule heat and cuts the tissue [45]. When high-frequency current is conducted through a narrow root canal, bubbles are generated by Joule heat, and the current density can increase between the bubbles and the root canal wall, possibly resulting in an instantaneous temperature rise. As a result, the water may rapidly evaporate, and the organic material on the root canal wall may be eliminated. This is a mechanism similar to that of the Er-YAG laser, but the details must await future elucidation.

In addition, high voltages may cause electrical discharges within air bubbles generated in the root canal. An electrical discharge within the bubble also generates a plasma with high antibacterial properties [46,47,48,49,50]. An analysis of bubble generation and short-term changes in the electric field due to the application of electricity may lead to a better understanding of its potential application to root canal treatment and possibly other fields, but many further studies are needed to elucidate the actual mechanism.

Cloudy or black root canal walls observed with an optical microscope were seen to have a lava-like morphology on SEM. Because no carbon was detected in this site by EDS, it might not have been carbonized but ashed. Because the surface morphology was lava-like and porous, the size and number of pores may have appeared cloudy or black with an optical microscope. Because the sample was thoroughly washed after cutting and before observation with an optical microscope, it is unlikely that the carbonized layer was removed during the subsequent preparation of the SEM specimen. However, because the relationship between surface morphology and color is unclear, detailed research is required in the future.

The fact that the cauterizing effect of electrical conduction was stronger with sodium hypochlorite than with physiological saline may have been due to the differences in the elements within the bubbles produced by heat generation, which for physiological saline would be steam bubbles produced by heat generation, but for sodium hypochlorite, there would have been a high OCl^−^ radical content. This is another topic for further investigations. In this experiment, voltages of 150 and 225 V were tried, but it may be possible to shorten the conduction time by further increasing the voltage. If the root canal becomes narrower or longer and the impedance is high, a higher voltage may be effective in increasing the current value and the heat generation. This is yet another topic for future study. Root canal treatment using high-frequency current has been studied for a long time, but it has been used to heat the tip of the electrode. However, Cassanelli et al. [51] showed that short-duration applications of high-voltage, high-frequency currents could be used to sterilize uninstrumented areas, using an effect similar to electroporation. Although this method can be used to achieve sterilization, dead bacteria remain, which may reduce the sealing ability of root canal fillings. Although the sterilization effect of the high-frequency current used in this study was not examined, it is possible that the bacteria and the extracellular matrix that constitute the biofilm can be extinguished by incineration due to high temperature.

The electrotome used for cutting and coagulating soft tissue produces thousands of volts at frequencies of 300–5000 kHz. Therefore, the voltage and frequency used in this experiment are considered to be extremely safe. A high temperature is generated in the narrow root canal by the application of electricity, but heat generation is limited to the root canal for a short period of time. Because the current spreads over a wide area in the periodontal ligament, it is expected that heat generation is significantly reduced in the periodontal tissue. In the case reports published thus far in which electricity had been applied to the root canal, no side effects suggestive of damage to the periodontal tissue have been reported [35,36]. Moreover, it is thought that the periodontal ligament does not retain heat due to blood flow [52]. However, long-term energization causes the root canal solution to accumulate heat, and the temperature rise in the external root surface due to heat dissipation may damage the periodontal tissue. In particular, because bone necrosis occurs at 47 °C [53], it is necessary to carefully verify the voltage and duration of application that do not damage the bone. Continuous energization time and solution replacement are also important considerations.

Mechanical cleaning is fundamental to root canal treatment, and the bacteria within the root canal are eradicated by irrigation with sodium hypochlorite and other disinfectants, but high-frequency electric current may provide a third method of eliminating pathogenic factors. High-frequency electric current should be able to achieve disinfection as long as a conductive liquid can permeate the root canal area that cannot be instrumented. This may lead to the cure of many cases of persistent apical periodontitis. In addition, it should be possible to incinerate and cause the pulp tissue in uninstrumented areas where the pulp cannot be mechanically removed, such as lateral root canals, to disappear by applying electricity at the pulpectomy, which can be expected to reduce residual pulpitis and postoperative pain [54]. However, the present study was conducted with a standardized and simulated root canal, and the cross-sectional shape of the root canal in human teeth is not circular or uniform in size. In addition, the simulated root canals were made from bovine teeth, which contain more organic material than human dentine. Therefore, whether organic material of all human root canal walls can be eliminated remains unclear. A future study should examine in detail the appropriate energization time and voltage for human teeth. In addition, the ability to seal the root canal after energization needs to be investigated in the future. There are many issues to be resolved, but high-frequency electric current has the potential to revolutionize root canal treatment.

## 5. Conclusions

Application of a high-frequency electric current of 520 kHz, 150 or 225 V, for 1 to 5 s to simulated root canals not in contact with the electrodes caused organic material to disappear from the root canal walls. This result was more pronounced when the solution in the root canal was sodium hypochlorite rather than physiological saline, and it also intensified with increasing voltage and conduction time.

## Figures and Tables

**Table 1:** Energy conditions for each group (*n* = 6 in each group).

	Root Canal Solution	Voltage	Conduction Time
Conduction group	Physiological saline	150	1
			5
		225	1
			5
	NaClO	150	1
			5
		225	1
			5
Control group	NaClO	-	-

## Data Availability

The data presented in this study are available from the corresponding author, T.S., upon reasonable request.
